# Novel compounds with antiangiogenic and antiproliferative potency for growth control of testicular germ cell tumours

**DOI:** 10.1038/sj.bjc.6605725

**Published:** 2010-06-15

**Authors:** B Nitzsche, C Gloesenkamp, M Schrader, M Ocker, R Preissner, M Lein, A Zakrzewicz, B Hoffmann, M Höpfner

**Affiliations:** 1 Department of Physiology, Charité – Universitätsmedizin Berlin, Campus Benjamin Franklin, Arnimallee 22, Berlin 14195, Germany; 2 Department of Urology, University of Ulm, Prittwitzstraße 43, 89075 Ulm, Germany; 3 Institute for Surgical Research, Philipps University Marburg, Baldingerstrasse, 35043 Marburg, Germany; 4 Berlin Institute for Urologic Research, Charité –Universitätsmedizin Berlin, Robert Koch -Platz 7, Berlin 10115, Germany

**Keywords:** antiangiogenesis, cell cycle, testicular germ cell tumour, tyrosine kinase inhibitor, vascular endothelial growth factor receptor-2 (KDR)

## Abstract

**Backround:**

Testicular germ cell tumour (TGCT) is the most common cause of death from solid tumours in young men and especially for platinum-refractory patients novel treatment approaches are urgently needed. Using an *in silico* screening approach for the detection of novel cancer drugs with inhibitory effects on the tyrosine kinase activity of growth factors (e.g., VEGFR, PDGFR), we identified two compounds (HP-2 and HP-14) with antiangiogenic and antiproliferative potency, which were evaluated in endothelial cell models and TGCT cells.

**Results:**

HP-2 and HP-14 effectively inhibited the growth of VEGFR-2-expressing TGCT cell lines (Tera-1, Tera-2 and 2102EP) and endothelial cell models, while they failed to supress the growth of VEGFR-2-lacking tumour cells. cDNA-microarrays revealed an inhibition of the expression of several growth factor receptors and related signal transduction molecules. Vascular endothelial growth factor (VEGF)-induced cell migration was also potently inhibited. Cell cycle-regulating proteins such as p21 and p27 were upregulated, leading to an S-phase arrest. Additional *in vivo* evaluations confirmed the antiangiogenic potency and good tolerability of the novel substances.

**Conclusion:**

Our data show that the identified novel compounds inhibit the growth of TGCT cells and decrease angiogenic microvessel formation. The mode of action involves cell cycle arresting effects and changes in the expression pattern of several angiogenic genes. The novel compounds may qualify as new candidates for targeted treatment of TGCT and merit further evaluation.

Testicular germ cell tumour (TGCT) is the most common malignancy in the group of young men between 20 and 40 years with increasing incidence during the last 30 years in the majority of industrialised countries (Huyghe *et al*, 2003). Approximately 95% of malignant tumours of the testis are germ cell tumours (Bosl and Motzer, 1997). Germ cell tumours are classified as seminomatous or nonseminomatous and originate from carcinoma *in situ*, also known as testicular intraepithelial neoplasia (Looijenga and Oosterhuis, 1999). Today >90% of all patients with metastatic germ cell cancer can be cured after receiving standard-dose, cisplatin-based combination chemotherapy (Kollmannsberger *et al*, 2006). However, a small group of patients who relapse after cisplatin-based chemotherapy or from complete remission have a poor prognosis (Hussain *et al*, 2008). Therefore, it is very important to identify new treatment options for as much as to develop therapies with minimal side effects to improve the quality of life of these mostly young patients (Schrader *et al*, 2009).

Angiogenesis, the formation of new blood vessels, is a complex process that includes endothelial cell proliferation, vessel sprouting, vascular permeability and remodelling and maturation of emerging vessels. This process, mainly driven by the vascular endothelial growth factor VEGF/VEGFR-system, has a crucial role in tumour growth, development and progression (Loges *et al*, 2009). The necessary requirement for the expansion and metastasis of tumour cells is the connection to the vascular system of the surrounding tissue. This tumour angiogenesis will supply the tumour with sufficient oxygen and nutrients and is essential for tumour survival (Folkman, 1971). One of the most important signals for induction of angiogenesis is the VEGF. Vascular endothelial growth factor represents a family of five glycoproteins (VEGF-A–VEGF-E) in which VEGF-A (VEGF) is thought to be the most important angiogenic factor of this family. It binds to three structurally highly related tyrosine kinase receptors VEGFR-1 (Flt-1), VEGFR-2 (KDR/Flk-1) and VEGFR-3 (Flt-4) with specific functions: the binding of VEGF to VEGFR-1 leads to hematopoietic cell development, whereas VEGFR-3 is mainly involved in lymphatic endothelial cell development (Folkman, 1971; Ferrara *et al*, 2003). VEGFR-2 regulates the vascular endothelial cell development and is thought to be the primary receptor involved in angiogenesis. The expression of VEGFR-2 and VEGF is directly correlated with microvessel density and metastasis in many solid tumours (Boocock *et al*, 1995; Takahashi *et al*, 1996; Fox *et al*, 2004; Höpfner *et al*, 2008). This also holds true for germ cell tumours, in which the expression of VEGF correlates with increased angiogenic activity, disease progression and increased tumour microvessel density (Viglietto *et al*, 1996). Inhibition of the VEGF/VEGFR system has already become a clinically relevant strategy for innovative treatment of several urological tumours (Krause and Van Etten, 2005; Chieffi, 2007). However, information on the suitability for treatment of germ cell tumours remains to be determined.

In our study, we searched for novel antiangiogenic TKIs by using an *in silico screening* approach to identify yet unknown compounds with putative antiangiogenic and antiproliferative properties. The clinically relevant VEGFR TKI vatalanib was used as lead structure (Wood *et al*, 2000). An ‘inhouse’ database with approximately four million compounds was verified for two- and three-dimensional similarity to vatalanib. The search revealed 15 compounds with different antiproliferative and antiangiogenic potencies. Table 1 shows the two-dimensional similarities of 15 identified novel compounds. The purpose of this study was to determine and analyse the antiangiogenic, antimigratory and antiproliferative potency of the two most potent antiangiogenic and antiproliferative compounds, named HP-2 and HP-14. Evidence for the specific VEGFR-2 inhibition by the novel compounds in terms of binding properties and VEGFR-2-related inhibition of tumour cell growth was already shown in a previous study (Schmidt *et al*, 2008).

## Materials and methods

### Cell lines

The human testicular carcinoma cell line Tera-1 and Tera-2 (ATCC No. HTB-105, HTB-106) and the human renal cell carcinoma cell line A498 (ATCC No. HTB-44) were cultured in RPMI 1640 medium (Biochrom AG, Berlin, Germany) supplemented with 10% fetal bovine serum, 100 U ml^–1^ penicillin and 100 *μ*g ml^–1^ streptomycin. The human testicular carcinoma cell line 2102EP (Wang *et al*, 1981) (kindly provided by F Honecker, Hamburg, Germany) were cultured in DMEM/Ham's F-12 (1 : 1) medium (Biochrom AG) supplemented with 10% fetal bovine serum, 100 U ml^–1^ penicillin and 100 *μ*g ml^–1^ streptomycin. The endothelial cell line EA.hy926 (Edgell *et al*, 1983) was maintained in DMEM (Biochrom AG) with 10% fetal bovine serum, 100 U ml^–1^ penicillin and 100 *μ*g ml^–1^ streptomycin. Human umbilical vein endothelial cells (HUVECs) were isolated as described (Chlench *et al*, 2007). Endothelial Cell Basal Medium (PromoCell, Heidelberg, Germany) was supplemented with the SupplementPack MV (PromoCell). HUVEC cells were used at passages 1–2. The cultures were maintained at 37 °C in a humidified atmosphere of 5% CO_2_. Culture media was changed every second day and once a week the cells were passaged using 1% Trypsin/EDTA.

### Drugs

Vatalanib was obtained from LC Laboratories (Woburn, MA, USA). HP-2 and HP-14 were purchased from Ambinter (Paris, France). Stock solutions were prepared in DMSO, stored at −20 °C and diluted to the final concentration in fresh media before each experiment. In all experiments, the final DMSO concentration was <0.2%.

### Measurement of growth inhibition

Drug-induced changes in cell numbers were evaluated by crystal violet staining, as described (Gillies *et al*, 1986). In brief, cells in 96-well plates were fixed with 1% glutaraldehyde and stained with 0.1% crystal violet. The unbound dye was removed by washing with water. Bound crystal violet was solubilised with 0.2% Triton-X-100. Light extinction that increases linearly with the cell number was analysed at 570 nm using an ELISA-Reader.

### Western blotting

Western blotting was performed as described (Höpfner *et al*, 2004). In brief, whole-cell extracts were prepared by lysing cells with RIPA buffer. Lysates containing 30 *μ*g protein was subjected to gel electrophoresis. Proteins were transferred to PVDF membranes by electroblotting for 1.5 h. Blots were blocked in 5% skim milk powder solution (Merck, Darmstadt, Germany) for 1 h, and then incubated at 4 °C overnight with antibodies directed against ERK1/2 and pERK1/2 (1 : 500 or 1 : 1000, Santa Cruz Biotechnology, Santa Cruz, CA, USA) as well as with p21^waf1/cip1^ (1 : 200 Cell Signaling, Danvers, MA, USA) and p27^Kip1^ (1 : 2000 Cell Signaling). After incubation with horseradish peroxidase-coupled anti-IgG antibodies (1 : 10 000, Amersham, Uppsala, Sweden) at room temperature for at least 1 h, the blot was developed using enhanced chemiluminescent detection (Amersham) and subsequently exposed to Hyperfilm ECL film (Amersham) for 0.5–5 min.

### Reverse transcription polymerase chain reaction (RT–PCR)

Total RNA was extracted using RNeasy Mini Kit following the manufacturer's instructions (Qiagen, Hilden, Germany). The concentration and purity were measured by absorption spectrophotometry at 260 and 280 nm. The cDNA was synthesised from 2 *μ*g of total RNA using the Superscript RT kit (Invitrogen, Carlsbad, CA, USA) according to the manufacturer's protocol. Reverse transcription–polymerase chain reaction was carried out in a total volume of 50 *μ*l containing 200 nM of each Primer, 200 *μ*M dNTP's (Invitrogen), 1.5 mM MgCl_2_ and 2 U aTaq DNA-Polymerase (Promega, Madison, WI, USA). The PCR was performed in a Peltier thermal cycler (PTC-200, MJ-Research, Watertown, MA, USA) with the primers and conditions indicated in Table 2 (Dias *et al*, 2000; Basciani *et al*, 2002; Chung *et al*, 2005).

### Migration assay

EA.hy926 endothelial cells were grown to confluence in six-well plates. After serum starvation for 24 h, the monolayer was scratched with a pipette tip along a ruler. Endothelial cell growth medium was replenished with the new medium containing 10 ng ml^–1^ VEGF-A (VEGF-A_165_ Sigma-Aldrich, Steinheim, Germany) and 10 *μ*M of HP-2 or HP-14, respectively. Images were taken with Kappa digital camera (Kappa opto-electronics, Gleichen, Germany) after 24 h of incubation at 37 °C in a humidified atmosphere (5% CO_2_). Cell migration was quantified by using Tscratch software (Gebäck *et al*, 2009).

### DNA-microarray

Total cellular RNA was extracted from cells using ArrayGrade Total RNA Isolation Kit (SABiosciences, Frederick, MD, USA). RNA concentration was measured by absorption spectrophotometry (GeneQuant, Biochrom, Cambridge, UK). Using the True-Labeling AMP 2.0 amplification kit (SABiosciences), the mRNA was reversely transcribed into cDNA and converted to biotin-labeled cRNA using biotin-16-UTP (Roche, Mannheim, Germany) by *in vitro* transcription. cRNA samples were purified with an ArrayGrade cRNA cleanup kit (SABiosciences). Thereafter, the probes were hybridised to the pretreated Oligo GEArray Human Angiogenesis arrays (OHS-024, SABiosciences), which cover 113 angiogenesis-related genes plus controls or to Human Cancer Pathway Finder arrays (OHS-033, SABiosciences). After several washing steps, array spots binding cRNA were detected by chemiluminescence staining. Image acquisition was performed using X-ray films and a digital scanner. Spots were analysed and converted to numerical data by using the GEArray Expression Analysis Suite software (SABiosciences). Data evaluation included background correction (substraction of minimum value) and normalisation to reference genes. The cut off for upregulation was set at a 1.5-fold increase in the ratio of genes in the treated samples, whereas downregulation was determined as the 0.5-fold expression of genes in the treated samples.

### Cell cycle analysis by flow cytometry

Cell cycle analysis was performed by a modified method of Fried *et al* (1976). Cells were seeded at a concentration of 10^5^ cells ml^–1^ and treated with 10 *μ*M HP-14 for 24 h. Cells were then washed with PBS and fixed in PBS/formaldehyde 2% (vol/vol) on ice for 30 min. Afterwards cells were incubated in ice cold ethanol/PBS (2 : 1 vol/vol) overnight at −20 °C and pelleted. Resuspension in PBS containing 40 *μ*g ml^–1^ RNase A followed. After incubation for 30 min at 37 °C, cells were pelleted again and resuspended in PBS containing 50 *μ*g ml^–1^ propidium iodide. Cells were then analysed on a FACSCalibur flow cytometer using CellQuestPro Software (BD Biosciences, Heidelberg, Germany) and FlowJo Software (Tree star, Ashland, OR, USA).

### Chick chorioallantoic membrane (CAM) angiogenesis assay

Fertilised chicken eggs (Lohmann Tierzucht, Cuxhaven, Germany) were bred in an incubator at 37 °C in constant humidity for 3 days. After day 3, a square window was cut into the shell of each egg, and 5 ml of albumen was removed to allow detachment of the developing chorioallantoic membrane from the shell. The window was sealed with tape, and the eggs were bred in the incubator for additional 7 days. On day 11, the tapes were removed and the CAMs were treated with the different compounds as described (Ribatti *et al*, 1997). In brief, a small ring was placed onto the CAM and either 100 *μ*l of PBS (negative control) or 100 *μ*l of PBS containing HP-2 and HP-14 were added. After 48 h of incubation, the CAMs were examined and *in vivo* pictures were taken using a stereomicroscope equipped with a Kappa digital camera system. For more detailed investigations the CAMs were fixed with 4% paraformaldehyde, dissected and transferred to glass slides and analysed under the microscope (Zeiss Axioplan, Carl Zeiss, Oberkochen, Germany) equipped with a MBF Bioscience camera system (MBF Bioscience, Williston, VT, USA). The response to drug treatment was assessed by examining the alterations of the CAM differing from the controls.

## Results

### VEGFR expression

**Figure 1:**
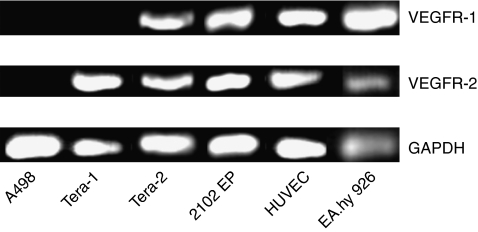
Expression of VEGFR-1 and VEGFR-2 in endothelial and urologic tumour cells. The TGCT cell lines Tera-1, Tera-2 and 2102EP show a strong expression of VEGFR-2 and a weak expression of VEGFR-1. By contrast urologic A498 tumour cells did not express any of the two receptors. Both endothelial cell models (EA.hy926 and HUVEC) showed the expected robust expression of VEGFR-1 and -2.

The expression of VEGFR-1 and VEGFR-2 was examined in endothelial cells (HUVEC and EA.hy926) and in the urologic tumour cell lines Tera-1, Tera-2, 2102EP and A498. Reverse transcription–PCR revealed a robust expression of VEGFR-2 in TCGT cells (Tera-1, Tera-2 and 2102EP) and in the two endothelial cell models. However, in the additionally tested renal cell carcinoma cells (A498) no appreciable expression of VEGFR-2 was detected. No expression of VEGFR-1 was detected in A498 and Tera-1 cells (Figure 1).

### Growth inhibitory effects

**Figure 2:**
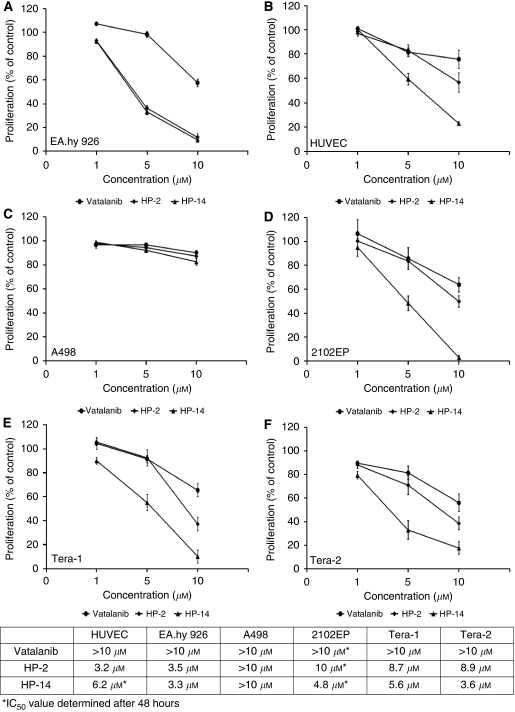
Antiproliferative effects of HP-2, HP-14 and vatalanib measured after 48 or 96 h. IC_50_ values of HP-2, HP-14 and vatalanib were determined in EA.hy926 (**A**), HUVEC (**B**), A498 (**C**), 2102EP (**D**), Tera-1 (**E**) and Tera-2 (**F**) cells. HP-2 and HP-14 exerted marked growth inhibitory effects in VEGFR-expressing TGCT cells Tera-1, Tera-2, 2102EP and in endothelial cell models in a time- and dose-dependent manner. No appreciable growth inhibition was observed in VEGFR-2-lacking A498 cells. Data are given as percentage of control, *value* mean; *bars*, s.e. (*n*=4).

To determine the growth inhibitory effects of HP-2 and HP-14 on tumour and endothelial cells, crystal violet staining was performed after 48 h of continuous incubation with rising concentrations of either compound. Both, HP-2 as well as HP-14 led to time- and dose-dependent growth inhibition of HUVEC, EA.hy926 and TGCT cells (Tera-1, Tera-2 and 2102EP) of up to >80%. Interestingly, VEGFR-lacking A498 cells did not respond to HP-treatment (Figure 2). Compared with the growth inhibitory effects of the lead structure vatalanib, the antiproliferative effects of our novel compounds were much more pronounced, especially in the TGCT cells.

Unspecific cytotoxicity of HP-2 and HP-14 was excluded by measurement of LDH-release into the supernatant of treated cells. Neither endothelial cells nor TGCT cells showed an increase in LDH release >1% as compared with untreated control cells, revealing that unspecific cytotoxicity does not account for the observed antiproliferative effects of the two compounds (data not shown).

### Gene expression profiling

To shed light on the pathways modulated by the novel compounds two different cDNA-microarrays were performed. The *human angiogenesis array* was performed with endothelial cells (HUVEC) and TGCT cells (Tera-1) to profile the expression of 113 genes involved in angiogenesis after treatment with HP-14 (15 *μ*M for 48 h). The *human cancer pathway finder array* was used to determine the expression pattern of genes involved in cancer relevant pathways such as transformation and tumourigenesis of TGCT cells (Tera-1). On HP-14 treatment up- or downregulation of 35 genes involved in angiogenesis were detected in HUVEC cells (Table 3). The angiogenesis-promoting genes Tie-2 and IL8 showed a strong downregulation, while the antiangiogenic regulator endostatin was markedly upregulated.

In Tera-1 cells, HP-14 induced the modulation of 40 genes responsible for cellular functions such as growth, signal transduction, cell cycle regulation and angiogenesis (Table 4). Genes encoding proteins regulating the progression of the cell cycle (CDK2, CDC25A) were strong suppressed and the major cell cycle inhibitor CDKN1A was elevated. In addition, a suppression of the proangiogenic growth factors PDGFA, PDGFB and HTATIP2 was detected, as well as an elevation of the angiogenic inhibitor endostatin.

Both, in endothelial as well as in TGCT cells HP-14 induced a marked downregulation of VEGFR-2 (KDR/Flk-1). Moreover, in Tera-1 cells the VEGFR-2 downstream signalling transduction molecule AKT1 was suppressed, suggesting a functional role of HP-14 in these processes.

The Tables 3 and 4 summarise the findings on up- and downregulation of angiogensis- and cancer pathway-specific genes of endothelial and TGCT cells, respectively.

### MAPK-inhibition by novel compounds

**Figure 3:**
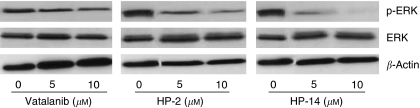
Treatment of EA.hy926 cells with HP-2 and HP-14 led to a dose-dependent decrease in ERK1/2 phosphorylation, whereas the expression of total ERK1/2 protein was not affected. Compared with vatalanib the inhibitory effect of HP-2 and HP-14 was more pronounced.

Inhibition of ERK1/2 phosphorylation was examined in EA.hy926 cells. Cells were incubated with 0, 5 and 10 *μ*M of HP-2 or HP-14 for 24 h. Both compounds inhibited the phosphorylation of ERK1/2 (Figure 3), whereas the total amount of ERK protein remained unaffected. Maximal effects were observed with 10 *μ*M of HP-14.

At equimolar concentration HP-2 and HP-14 show stronger inhibition of ERK1/2 phosphorylation as compared with vatalanib. Comparable results were obtained in the TGCT cell lines (data not shown).

### Drug-induced cell cycle arrest

**Figure 4:**
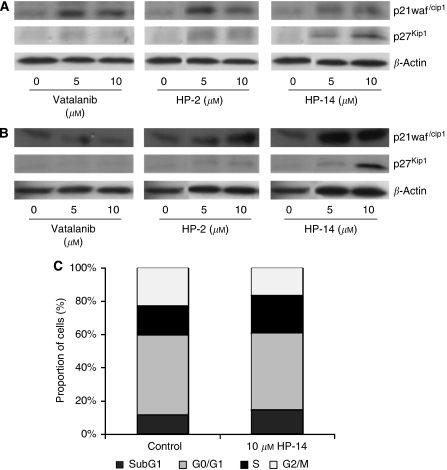
Expression of the cyclin-dependent kinase inhibitors (p21^waf/Cip1^, p27^Kip1^) was examined by western blotting. Incubation of Tera-1 (**A**) and EA.hy926 (**B**) with HP-2, HP-14 and vatalanib resulted in an increase in the expression of the cell cycle inhibitors p21^waf/Cip1^ and p27^Kip^. FACS analysis of HP-14 (10 *μ*M) treated Tera-1 cells revealed an arrest in the S-phase of the cell cycle, with a concomitant decrease in cells in the G2/M phase and no significant increase in apoptotic cells in the SubG1 population (**C**).

Expression of the two major cell cycle regulators, the cyclin-dependent kinase inhibitors p21^waf/cip1^ and p27^Kip1^, were examined by western blotting. Incubation of Tera-1 and EA.hy 926 cells with HP-2, HP-14 or vatalanib for 24 h resulted in an increase in p21^waf/cip1^ and p27^Kip1^ expression, suggesting that the mode of action of the HP-substances involves cell cycle arresting effects (Figures 4A and B). To confirm these findings flow cytometric cell cycle analyses was performed on Tera-1 cells. In this study, HP-14 treatment led to an arrest of cells in the S-phase of the cell cycle, accompanied by a respective decrease in the proportion of cells in the G2-phase, whereas no significant increase in apoptotic cells in the SubG1 population was detected (Figure 4C).

### Effects on cell migration

**Figure 5:**
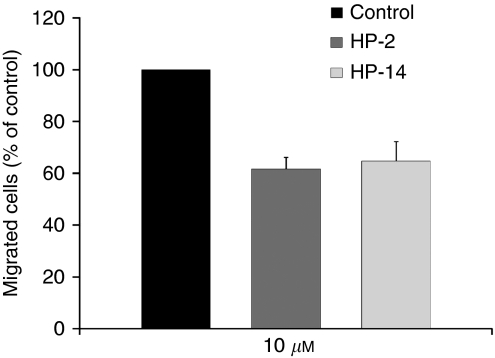
Antimigratory effects of HP-2 and HP-14 were analysed by performing scratch wound assays. Serum-starved cell monolayers were scratched with a pipette tip and migration was stimulated by application of VEGFA (10 ng ml^–1^). Compared with control the migration of HP-treated cells was reduced by ∼40%. Data are given as percentage of control (means±s.e.m. of three independents experiments).

Cell migration is necessary for endothelial angiogenesis as well as for cancer cell invasion and metastasis. Performing scratch wound assays we determined the antimigratory effects of HP-2 and HP-14 on endothelial cell migration. EA.hy926 cell monolayers were serum starved for 24 h. Thereafter the monolayers were scratched with a pipette tip and scratch closure was stimulated by VEGF (10 ng ml^–1^). HP-2 and HP-14 pretreated cells showed a decreased migration as compared with VEGF-stimulated control cells. The migratory inhibition of HP-compounds amounted to a maximum of 38% after 24 h, as determined by TSCRATCH (Figure 5).

### *In vivo* evaluation of HP-2- and HP-14-induced inhibition of angiogenesis

**Figure 6:**
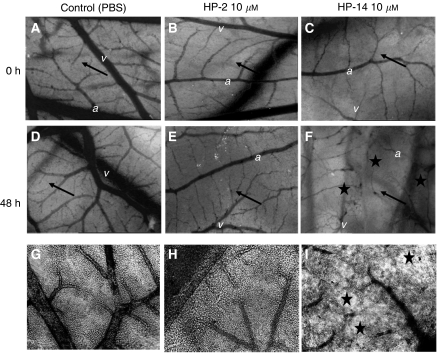
HP-2- and HP-14-induced vasodegeneration of the developing CAM of fertilised chicken eggs. Upper panel (**A**–**C**) depicts CAMs before treatment. In the middle panel, effects of 48 h of treatment with PBS (control) (**D**), HP-2 (10 *μ*M) (**E**) or HP-14 (10 *μ*M) (**F**) are shown. (*a*=artery and *v*=vein). The lower panel (**G**–**I**) shows microscopic pictures taken from dissected CAMs after treatment. In untreated and control CAM's (**A**–**D**), the vascular network consisted of a continuously perfused capillary plexus (*star*) and larger vessels (*arrow*). HP-2 and HP-14 induces a degeneration of the vascular network, which was identified as unperfused areas and changes in the small supplying vessels.

Antiangiogenic effects of HP-2 and HP-14 on microvessel formation of the developing CAM was assessed after 48 h of incubation. The capillary plexus and the immature larger supplying vessels (arteries and veins) were visible at the beginning of the experiment (Figures 6A–C). After 2 days, the PBS-treated control CAM (Figure 6D) showed mature large vessels (*arrow*) and a pronounced capillary plexus. By contrast, treatment with HP-2 (10 *μ*M) led to huge non-perfused areas of the CAM. Especially the smaller supplying vessels were influenced by HP-2 treatment (Figure 6E), whereas no obvious influence was observed on the capillary plexus (*star*). HP-14 had the strongest effect on the CAM vasculature. HP-14 (10 *μ*M, 48 h) led to an increase in non-perfused areas and to an obvious degeneration of the vasculature and the capillary plexus (Figures 6F and I).

## Discussion

Regardless of the generally successful platinum-based treatment possibilities for patients with metastatic testicular germ cell cancer, there are still subgroups of patients whose prognosis is poor. For instance, in patients with strongly reduced initial creatinine clearance adequate high-dose chemotherapy is problematic or even impossible because of the increasing risk of a further loss of renal function. Another problem is the poor response of recurrent tumours to second-line therapy and the dose limitation, leading to discontinuation of second salvage treatment with high-dose polychemotherapy before achieving the eradication of the advanced tumours. So in case of patients with primary platinum-resistant and/or recurrent tumours or those that are unable to undergo systemic cisplatin-based chemotherapy, alternative treatment are urgently needed (Lorch *et al*, 2007; Schrader *et al*, 2009).

Searching for novel non-platinum-based treatment approaches, we analysed and characterised the antiangiogenic and antiproliferative effects of two novel small molecule inhibitors, HP-2 and HP-14. The compounds were derived from *in silico* screenings using the clinically relevant VEGFR tyrosine kinase inhibitor vatalanib as the lead structure.

The rationale for choosing a VEGFR tyrosine kinase inhibitor as a lead structure was deduced from findings that proangiogenic VEGF is often increased in patients with TGCTs and increases metastatic potential of TGCTs (Fukuda *et al*, 1999; Aigner *et al*, 2003; Bentas *et al*, 2003). Moreover, the expression of VEGFR-2, which is the most important angiogenic growth factor receptor, has been implicated in the pathogenesis of testicular germ cell (Jones *et al*, 2000; Devouassoux-Shisheboran *et al*, 2003). Inhibition of the VEGF/VEGFR system has already become a clinically relevant strategy for treatment of some urological cancers, especially those refractory to the standard chemotherapy (Voigt *et al*, 2006; Fenner *et al*, 2008; Castillo-Ávila *et al*, 2009), whereas TGCTs are not systematically analysed in this respect.

In a recent phase II study, the multikinase inhibitor sunitinib blocking the tyrosine kinase activity of PDGFR, VEGFRs, c-kit and RET showed good tolerability in patients with multiply relapsed or refractory GCT. However, the clinical outcome was rather poor. Nevertheless, the observed decline of angiogenic tumour markers during the treatment suggested that pathways inhibited by sunitinib, such as the VEGF-pathway may be of particular importance for GCT biology. As these angiogenic pathways were presumably not enough suppressed by sunitinib – at least at the doses used in this trial (Feldman *et al*, 2009), we searched for a more potent and specific VEGFR-inhibitor, which may show a more pronounced antiangiogenic effect in GCT.

In our study, we could show that the two novel compounds, HP-2 and HP-14, strongly inhibit the proliferation of VEGFR-positive TGCT cells in a dose-dependent manner. Both agents also inhibited the proliferation of endothelial cells with IC_50_ values that were below those of vatalanib. VEGFR specificity of the compounds was shown in experiments with VEGFR-negative renal cell carcinoma cells, which did not respond to treatment with either vatalanib or HP-compounds.

To examine the underlying molecular events of the novel compounds, we profiled transcriptional changes in both TGCT cells as well as in endothelial cells by cDNA microarrays. HP-14 regulated a variety of genes, most of which could be ascribed to cellular functions such as growth, signal transduction and regulation of the cell cycle. On HP-treatment a significant reduction in VEGFR-2 expression was observed in both Tera-1 and HUVEC cells. In addition, the expression of AKT-1 (protein kinase B), a downstream signal transduction molecule of VEGFR-2 was also detected (Takano *et al*, 2008). Earlier findings by Anderson *et al* showed the cell proliferative effects of AKT-1, which can be activated by VEGFR-2 through protein kinase-C-dependent signalling (Anderson *et al*, 2008). Furthermore, HP-14 treatment also inhibited the expression of other angiogenic growth factors such as PDGFA, PDGFB or FGF-2, each of them being implicated in the promotion of testicular germ cell cancer (Peltomäki *et al*, 1991; Bentas *et al*, 2003). Another angiogenic factor that was suppressed by HP-14, was the HIV-1 Tat interactive protein (HTATIP2). This proangiogenic growth factor binds and activates VEGFR-2, thereby mediating cell proliferation, cell migration and cell survival (Albini *et al*, 1996). These data show that angiogenesis reflected by the serum concentrations of growth factors have a functional role in tumour growth of testicular germ cell cancer and that inhibition of these factors may thus be a promising treatment option.

HP-14 also increased the expression of antiangiogenic genes such as the angiogenesis inhibitor endostatin (COL18A1). Endostatin is a protein that is supposed to neutralise many VEGF-A-induced effects such as VEGF-induced endothelial cell migration, neovascularisation and vascular permeability (Yamaguchi *et al*, 1999; Takahashi *et al*, 2003). Thus, the upregulation of endostatin in endothelial cells during HP-14 treatment could be an explanation of antiangiogenic potency of this novel compound.

The antiproliferative mode of action of the novel compounds also involved cell cycle-regulating effects in TGCT cells. Defective function of cell cycle regulators is a main cause for tumour development and progression. For example, the cell cycle promoter cyclin D2 is frequently overexpressed in TGCTs, while cell cycle inhibitors, such as p21 are frequently suppressed (Chaganti and Houldsworth, 2000). Successful therapeutic strategies will thus have to balance or bypass this impaired signalling. In our study, we could show that treatment of TGCT cells with HP-compounds raised the expression of the cell cycle-inhibiting molecule p21, which resulted in an S-phase arrest of the cell cycle. Our observation that HP-treatment-induced S-phase arrest and p21 overexpression is in agreement with a previous report that transduction of the p21 gene resulted in an S-phase arrest (Ogryzko *et al*, 1997; Radhakrishnan *et al*, 2004; Zhu *et al*, 2004). Surprisingly, we also observed an increase in p27 expression on HP-treatment. In general, p27 is associated with an arrest in the G1-phase of the cell cycle. Nevertheless, p27 has also been shown to induce S-phase arrest of human hepatocellular carcinoma cells (Fuke *et al*, 2007). So far, the exact mechanism underlying p27-induced S-phase arrest remains to be examined. However, Sa and Stacey recently showed that growth signalling pathways, such as the AKT-pathway can regulate p27 expression in a cell cycle phase-independent manner. AKT inhibition led to an increase in p27 expression of cells that were arrested in the S-phase (Sa and Stacey, 2004). In our gene array experiments, we also observed an inhibition of the AKT because of HP-14 treatment. Thus, it is at least feasible that the S-phase arrest of HP-treated TGCT cells is mainly induced by an increase in p21, whereas the concomitantly observed AKT inhibition might lead to increasing levels of p27 of S-phase arrested cells. However, further investigations will have to clarify the exact role of p27 during HP-induced cell cycle arrest of TGCT cells.

The p21 expression is also correlated with a reduction of the cell cycle-controlling phosphatase protein cdc25A (de Oliveira *et al*, 2009), a protein that was found to be downregulated by the novel HP-compounds in TGCT cells. Suppression of cdc25A is implicated in the inhibition of tumour growth-promoting MAPK activity (Wang *et al*, 2008). The MAPK pathway protein ERK1/2 (extracellular signal-regulated kinase 1/2) is activated by VEGFR-2 (Rubinfeld and Seger, 2005; Narasimhan *et al*, 2009). Corresponding to the postulated VEGFR-specific action of the HP-compounds, we could show a decrease in ERK1/2 activity after HP-treatment.

Endothelial cell migration is one of the key characteristics in VEGFR-2-mediated angiogenesis (Shibuya, 2006). In this study, we could show that HP-2 and HP-14 both inhibited endothelial cell migration and suppress *in vivo* neovascularisation in a chorioallantoic membrane assay. Further evaluations will have to clarify the compatibility of the novel HP-compounds *in vivo*. In this respect, it is noteworthy that preliminary studies on healthy mice showed promising tolerability of the HP-substances (50 mg kg^–1^) when given in 48 h intervals for seven days. Treated mice (*n*=7) did neither show weight loss or any altered behaviour. Histological examination of kidney, spleen, lung and liver of the dispatched animals did not show abnormalities or signs of inflammatory infiltrations (data not shown).

In summary, our study showed that the identified novel compounds are able to potently block TGCT cell growth and angiogenesis *in vitro* and *in vivo*. The inhibitory effects of the novel compounds were even more pronounced than those of the clinically relevant VEGFR tyrosine kinase inhibitor, vatalanib, which was used as the lead structure for the identification of structurally related novel compounds with antiangiogenic and antiproliferative potency. The complex mode of action of HP-compounds involved cell cycle-arresting effects as well as a shifting of the balance of pro- and antiangiogenic genes towards angiogenic suppression and antiangiogenic stimulation (Baeriswyl and Christofori, 2009). The fact that only the growth of VEGFR-2-expressing endothelial and TGCT cells was markedly inhibited, suggests that VEGFR-2 is the major target of the novel HP-compounds. On the basis of our findings, we think that the presented compounds may become promising candidates for innovative approaches in TGCT treatment and warrant further evaluation.

## Figures and Tables

**Table 1:**
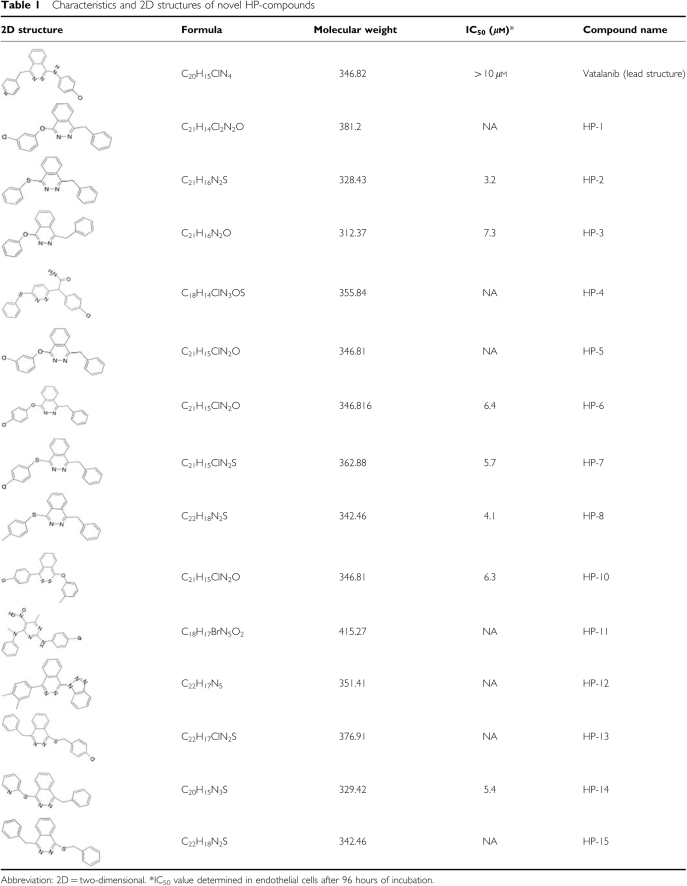
Characteristics and 2D structures of novel HP-compounds

**Table 2:** RT–PCR primer used for the detection of VEGF receptors

**Genes**	**Primers (5′-3′)**	**Gene bank accession number**	**Product size (bp)**	**Denaturing temperature and time (s)**	**Annealing temperature and time (s)**	**Extension temperature and time(s)**	**Number of cycles**
VEGFR-1	F:ATTTGTGATTTTGGCCTTGC	NM_002019	550	94 °C (60)	65 °C (45)	72 °C (120)	35
	R:CAGGCTCATGAACTTGAAAGC						
VEGFR-2	F:GTGACCAACATGGAGTCGTG	NM_002253	660	94 °C (60)	65 °C (45)	72 °C (120)	35
	R:CCAGAGATTCCATGCCACTT						
GAPDH	F:CCTGACCTGCCGTCTAGAAA	NM_002046	276	94 °C (15)	55 °C (30)	68 °C (60)	35
	R:TACTCCTTGGAGGCCATGTG						

Abbreviations: RT–PCR=reverse transcription polymerase chain reaction; VEGF=vascular endothelial growth factor.

**Table 3:** Genes regulated in HUVEC cells in response to HP-14 15 *μ*M for 48 h predicted by human angiogenesis array

**Symbol**	**Fold**	**Product**
*Downregulated genes*		
ANPEP	0.48	Alanyl aminopeptidase
EPAS1	0.48	Endothelial PAS domain protein 1
EGF	0.46	Epidermal growth factor
EFNA2	0.45	Ephrin-A2
KDR	0.44	Kinase insert domain receptor (VEGFR-2)
NRP1	0.37	Neuropilin 1
PLAU	0.32	Plasminogen activator, urokinase
TEK	0.25	TEK tyrosine kinase, endothelial
CCL11	0.23	Chemokine ligand 11
IL8	0.21	Interleukin 8
ECGF1	0.19	Endothelial cell growth factor 1 (platelet-derived)
TNNT1	0.17	Troponin T type 1
TIMP3	0.12	TIMP metallopeptidase inhibitor 3
NOTCH4	0.11	Notch homolog 4
EFNA1	0.10	Ephrin-A1
TNFRSF12A	0.09	Tumor necrosis factor receptor superfamily, member 12A
TIMP2	0.08	TIMP metallopeptidase inhibitor 2
TGFA	0.07	Transforming growth factor, α
VEGFA	0.06	Vascular endothelial growth factor A
EFNA3	0.04	Ephrin-A3
PGF	0.04	Placental growth factor, vascular endothelial growth factor-related protein
AKT1	0.04	V-akt murine thymoma viral oncogene homolog 1
PDGFB	0.03	Platelet-derived growth factor β polypeptide
IL1B	0.01	Interleukin 1, β
		
*Upregulated genes*		
ANPEP	1.64	Alanyl aminopeptidase
COL18A1	1.96	Collagen, type XVIII, α 1
IL6	2.19	Interleukin 6
EDG1	2.23	Endothelial differentiation, sphingolipid G-protein-coupled receptor, 1
VEGFB	2.29	Vascular endothelial growth factor B
TIE1	2.37	Tyrosine kinase with immunoglobulin-like and EGF-like domains 1
MMP2	2.41	Matrix metallopeptidase 2
TIMP1	2.45	TIMP metallopeptidase inhibitor 1
ENG	2.56	Endoglin
CXCL1	2.78	Chemokine ligand 1
EFNB2	2.93	Ephrin-B2

Abbreviation: HUVEC=human umbilical vein endothelial cell.

**Table 4:** Genes regulated in Tera-1 cells in response to HP-14 15 *μ*M for 48 h predicted by human angiogenesis and human cancer pathway array

**Symbol**	**Fold**	**Product**
*Downregulated genes*		
PDGFA	0.51	Platelet-derived growth factor α polypeptide
NFKB1	0.29	Nuclear factor of kappa light polypeptide gene enhancer in B cells 1
NFKBIA	0.23	Nuclear factor of kappa light polypeptide gene enhancer in B cells inhibitor
PDGFB	0.22	Platelet-derived growth factor β polypeptide
MTA1	0.22	Metastasis-associated 1
KDR	0.15	Kinase insert domain receptor (a type III receptor tyrosine kinase)
CXCL5	0.14	Chemokine (C-X-C motif) ligand 5
CDKN1B	0.14	Cyclin-dependent kinase inhibitor 1B (p27, Kip1)
MMP2	0.14	Matrix metallopeptidase 2
MTA2	0.13	Metastasis-associated 1 family
JUN	0.12	Jun oncogene
CDK2	0.07	Cyclin-dependent kinase 2
SERPINB5	0.07	Serpin peptidase inhibitor
ITGA4	0.06	Integrin, α 4
BRCA2	0.05	Breast cancer 2
EGFR	0.05	Epidermal growth factor receptor
AKT1	0.04	V-akt murine thymoma viral oncogene homolog 1
PRKDC	0.04	Protein kinase
HTATIP2	0.04	HIV-1 Tat interactive protein
FGF2	0.04	Fibroblast growth factor 2
CDC25A	0.03	Cell division cycle 25 homolog A
SERPINB2	0.03	Serpin peptidase inhibitor
		
*Upregulated genes*		
FGFR2	1.51	Fibroblast growth factor receptor 2
COL18A1	1.59	Collagen, type XVIII, 1
CCNE1	1.60	Cyclin E1
PNN	1.64	Pinin, desmosome-associated protein
TIE1	1.74	Tyrosine kinase with immunoglobulin-like and EGF-like domains 1
ITGB5	1.93	Integrin, β 5
CDK4	2.03	Cyclin-dependent kinase 4
NME1	2.03	Non-metastatic cells 1, protein (NM23A)
ITGB1	2.07	Integrin, β 1
BIRC5	2.11	Baculoviral IAP repeat-containing 5 (survivin)
TIMP1	2.11	TIMP metallopeptidase inhibitor 1
VEGFB	2.21	Vascular endothelial growth factor B
TNFRSF1A	2.23	Tumor necrosis factor receptor superfamily, member 1A
TNFRSF10B	2.24	Tumor necrosis factor receptor superfamily, member 10b
CDKN1A	2.27	Cyclin-dependent kinase inhibitor 1A (p21, Cip1)
PLAU	2.93	Plasminogen activator, urokinase
ITGA3	12.93	Integrin, α 3
TERT	24.59	Telomerase reverse transcriptase
